# Mechanisms, therapeutic implications, and methodological challenges of gut microbiota and cardiovascular diseases: a position paper by the ESC Working Group on Coronary Pathophysiology and Microcirculation

**DOI:** 10.1093/cvr/cvac057

**Published:** 2022-04-14

**Authors:** Dimitris Tousoulis, Tomasz Guzik, Teresa Padro, Dirk J Duncker, Giuseppe De Luca, Etto Eringa, Marija Vavlukis, Alexios S Antonopoulos, Themistoklis Katsimichas, Edina Cenko, Ana Djordjevic-Dikic, Ingrid Fleming, Olivia Manfrini, Danijela Trifunovic, Charalambos Antoniades, Filippo Crea

**Affiliations:** 1st Cardiology Department, National and Kapodistrian University of Athens, Vas. Sofias Avenue 114, 11527 Athens, Greece; Institute of Cardiovascular Medical Sciences, BHF Glasgow Cardiovascular Research Centre, UK; Sant Pau Institute for Biomedical Research, Barcelona, Spain; Department of Cardiology, Thorax Center, Erasmus MC, Rotterdam, the Netherlands; Division of Cardiology, Eastern Piedmont University, Novara, Italy; Institute of Cardiovascular Research, Amsterdam University Medical Center, Amsterdam, the Netherlands; University Ss. Cyril and Methodius, Skopje, North Macedonia; 1st Cardiology Department, National and Kapodistrian University of Athens, Vas. Sofias Avenue 114, 11527 Athens, Greece; 1st Cardiology Department, National and Kapodistrian University of Athens, Vas. Sofias Avenue 114, 11527 Athens, Greece; Department of Experimental, Diagnostic and Specialty Medicine, University of Bologna, Bologna, Italy; University Clinical Center of Serbia, Belgrade, Serbia; Centre of Molecular Medicine, Goethe University, Frankfurt, Germany; Department of Experimental, Diagnostic and Specialty Medicine, University of Bologna, Bologna, Italy; Cardiology Department, University of Belgrade, Belgrade, Serbia; RDM Division of Cardiovascular Medicine, University of Oxford, Oxford, UK; Department of Cardiology and Pulmonary Sciences, Catholic University of the Sacred Heart, Rome, Italy

**Keywords:** Coronary artery disease, Atherosclerosis, Gut microbiota, Trimethylamine N-oxide, Short-chain fatty acids

## Abstract

The human gut microbiota is the microbial ecosystem in the small and large intestines of humans. It has been naturally preserved and evolved to play an important role in the function of the gastrointestinal tract and the physiology of its host, protecting from pathogen colonization, and participating in vitamin synthesis, the functions of the immune system, as well as glucose homeostasis and lipid metabolism, among others. Mounting evidence from animal and human studies indicates that the composition and metabolic profiles of the gut microbiota are linked to the pathogenesis of cardiovascular disease, particularly arterial hypertension, atherosclerosis, and heart failure. In this review article, we provide an overview of the function of the human gut microbiota, summarize, and critically address the evidence linking compositional and functional alterations of the gut microbiota with atherosclerosis and coronary artery disease and discuss the potential of strategies for therapeutically targeting the gut microbiota through various interventions.

## Graphical Abstract

**Graphical Abstract:**
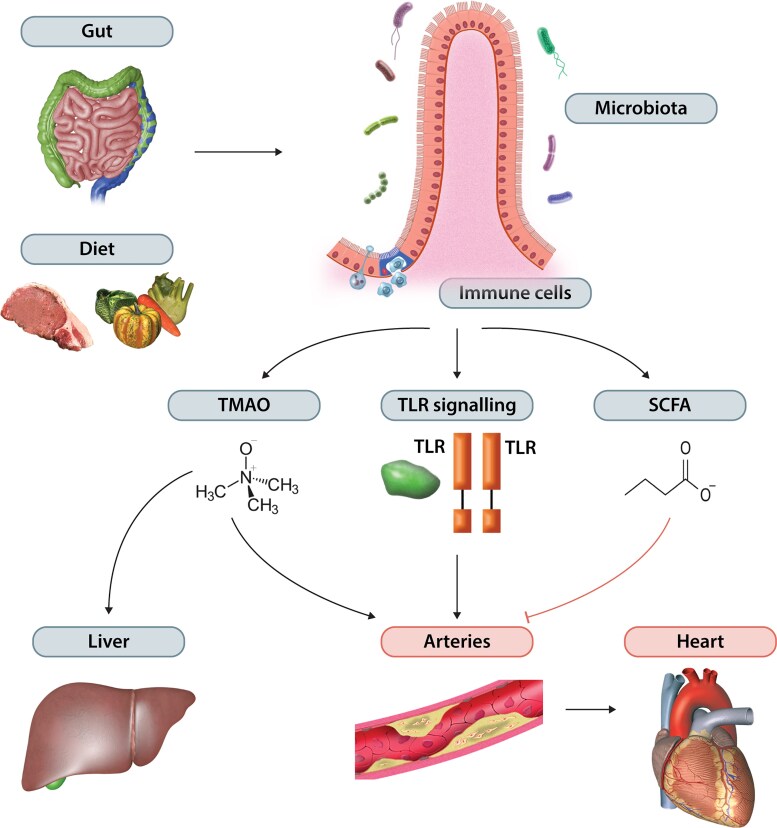
The pathophysiology of gut microbial associations with atherosclerosis. The figure depicts some of the key players at the organ, cellular, and molecular level, regarding the links between the gut microbiota and atherosclerosis. Diet has a profound effect on the gut microbiota, as it provides energy sources for bacterial fermentation. Undigested fibre is processed by bacteria-producing short-chain fatty acid (SCFA). Lower levels of SCFA have been linked to host inflammation, which aggravates atherosclerosis. Multiple bacterial species also produce trimethylamine (TMA) from choline, itself derived from dietary phosphatidylcholine. TMA is absorbed and metabolized to trimethylamine N-oxide in the liver and has been linked to the atherosclerotic process. Disruption of intestinal permeability may lead to bacterial lipopolysaccharide leaking into the bloodstream, initiating Toll-like receptor–mediated chronic systemic inflammation, and aggravating atherosclerosis. See text for details. Black arrows: Microbiota-related metabolites and host responses. Red arrows: Suggested proatherogenic effects.

## 1. Introduction

Cardiovascular disease (CVD), particularly coronary artery disease (CAD), still represents the leading cause of death worldwide^1^ despite effective progress in therapeutic interventions, such as early revascularization after acute coronary syndromes, lowering blood cholesterol levels, and inhibiting the renin-angiotensin-aldosterone system. Therefore, intense effort has been put into exploring and identifying new, therapeutically exploitable risk factors for atherosclerosis, to potentiate primary and secondary CAD prevention. Growing awareness of the influence of the human gut microbiota (GM) in the physiology of its host has led to the suggestion that it could contribute to the occurrence and development of atherosclerosis.^2^ Indeed, this microbial community living in the human intestinal tract can produce numerous metabolites that can enter systemic circulation and affect host health.^3^ Both metabolism-dependent and independent pathways have been proposed to explain the impact of the GM on atherogenesis.^4^ For example, the GM may exert pro-atherogenic effects via the synthesis of metabolites such as trimethylamine (TMA).^5^ Moreover, bacterial components such as lipopolysaccharides (LPS), found on the outer membrane of Gram-negative bacteria, can cross the host intestinal barrier, leak into the systemic circulation, and under certain circumstances contribute to low-grade chronic systemic inflammation, which by itself is a proatherogenic state.^6^

In this review, we briefly summarize existing knowledge on the GM and the tools developed for its compositional analysis, and we critically review the links between the GM and CAD. A glossary of terms is provided in *Table 1*.

**Table 1:** Glossary of terms

Term	Definition
Amplicon sequencing	The identification and amplification of a certain genetic sequence, e.g. regions of the 16S ribosomal RNA gene of the microorganisms in a human stool sample.
Alpha diversity	A term referring to individual characteristics of a microbial community (e.g. the number of different species).
Beta diversity	A concept of direct comparison of different microbial communities by a certain metric.
Commensalism	Co-existence of host and hosted organism, where only one of the two benefits.
Dysbiosis	A change in microbial composition, relative to an arbitrarily defined healthy state.
Metagenomics	The genetic analysis and direct identification of all genomes in a sample of matter (e.g. a stool sample).
Microbiome	All the genomes of all the microorganisms in a specific environment.
Microbiota	The community of microorganisms living in a specific environment.
Mutualism	A beneficial relationship for both the host and the hosted organism.
NGS	Modern DNA sequencing techniques.
Prebiotics	Indigestible food compounds that are fermented by and stimulate growth/activity of beneficial intestinal microorganisms (e.g. components of dietary fibre).
Probiotics	An accepted definition is ‘live microorganisms which when administered in adequate amounts confer a health benefit on the host’.
Synbiotics	The official definition is ‘a mixture comprising live microorganisms and substrate(s) selectively utilized by host microorganisms that confers a health benefit on the host’.

## 2. Methods of microbial analysis

Analysing the microbiome of diverse species and environments using next generation sequencing (NGS) techniques has significantly enhanced our understanding of metabolic, physiological, and ecological roles of environmental microorganisms (*Table 2*). The elucidation of the microbiome is not straightforward and is concerned with experimental conditions (i.e. sampling issues, sequencing errors, and genomic repeats) and computationally intensive and cumbersome downstream analysis (i.e. quality control, assembly, binning, and statistical analyses). Thorough reviews on the best practices and pitfalls associated with microbial analysis have been previously published.^7,8^

**Table 2:** Methods of microbial analysis

Method	Advantages	Disadvantages
Amplicon sequencing (marker gene analysis)	Simple and relatively cheap Large, publicly available datasets	Provides genus-level resolution at most No metabolic information (predictive algorithms required on top of analysis) No discrimination between live and dead organisms Multiple technical issues involved (choice of primers, amplification bias, other)
Metagenomics (metagenome analysis)	Species- and strain- level resolution Direct inferement of the relative abundance of metabolic genes No PCR bias	Expensive and complex Sensitive to contamination No discrimination between live and dead organisms
Metatranscriptomics (metatranscpriptome analysis)	Allows estimation of actively transcribing organisms Direct evaluation of microbial activity	Expensive and complex Sensitive to contamination Data skewed towards organisms with high transcription rates

PCR, polymerase chain reaction.

Gut microbial genetic analysis is generally performed by either amplicon sequencing or metagenomics.^7^ The most widely used target gene for bacterial identification in amplicon sequencing is the 16S *rRNA* gene, which encodes the 16S RNA component of the 30S small subunit of the prokaryotic ribosome. This method is relatively inexpensive and easily implemented, yielding genus-level taxonomic and relative abundance information on the GM sample.

Metagenomics is the direct genetic analysis of all genomes obtained from a given sample, without the need for cell cultures.^9,10^ This approach comprehensively catalogues all microbial genes, allowing the detection of genes that can provide information on molecular function and the metabolic profiling of a microbial community.^7^ Although this technology is more expensive and computationally challenging, it provides information on genetic diversity and potential microbial functions rather than simply on the taxonomic diversity of a community.^9,11,12^

Further options for GM analysis include metatranscriptomics, which uses RNA sequencing to profile transcription in microbiomes, providing information on gene expression and the active functional output of the microbiome. Sequencing microbial RNA provides better insight into the functional activity of a microbial community, though it is biased towards organisms with higher rates of transcription.^13^ Data from metagenomics and metatranscriptomics can be combined and compared by various bioinformatics tools.^7^ The exploration of additional -omics data, such as meta-proteomics and metabolomics, can also be performed. Newer developments are constantly under way, and long-read sequencing, a promising technique assessing genetic sequences of thousands of base pairs,^14^ has already been applied to canine faecal samples.^15^

## 3. Statistical analysis of microbial data

The two principal components of sample comparison in gut microbial research are alpha and beta diversity. Alpha diversity, often described as within-sample diversity, pertains to specific characteristics of a given microbial community, such as the number of different species. Biodiversity indices like the Shannon index can be calculated to average alpha diversity variation and compare it between groups of samples. Beta diversity, often described as between-sample diversity, refers to the direct comparison of different microbial communities. Similarity measures between samples are calculated, and the resulting data matrix is processed by multivariate mathematical tools, most popular of which are ANalysis Of Similarities (ANOSIM) and Permutational multivariate analysis of variance (PERMANOVA).^16,17^ These tools test whether microbial variation between *a priori* selected groups of samples is greater than variation within each group, yielding a decision for statistical significance. A framework for the analysis of multivariate ecological data has been thoroughly described.^18^

Differential abundance analysis between samples is demanding, with key issues being that relative abundance data are compositional (i.e. fractions or percentages, which when added must equal 1 or 100%), sparse, and non-normally distributed. Moreover, false-positive results are inevitable after hundreds of individual comparisons and must be controlled for. Analysis of compositions of microbiome (ANCOM) and its evolutions is one of the methods employed in this analysis.^19^ A recommended approach is to use the isometric log ratio transformation to render standard statistical tests valid in differential abundance testing.^7^ These issues also affect correlational analysis, necessitating specially designed statistical software, such as SParse InversE Covariance Estimation for Ecological Association Inference (SPIEC-EASI).^20^

A wide variety of bioinformatics software tools have been used to classify microbiota based on sequencing data. A study comparing 11 software tools for interpreting shotgun metagenomics found that they yielded different conclusions, highlighting the need to improve the accuracy of results by combining existing bioinformatics tools with different classification principles, thus controlling each software tool’s specific limitations and strengths.^21^ Machine learning algorithms may provide a solution, as these have been utilized to predict sample origin (e.g. patient vs. control subject) from microbiome data, among other predictions, such as future states of disease.^7^

## 4. Physiology and pathophysiology of the GM

### 4.1 Physiology of the microbial ecosystem in the human gut

The human GM has been estimated to comprise approximately 100 trillion microorganisms, including archaea, fungi, and viruses, but predominantly anaerobic bacteria belonging to several major bacterial phyla.^22^ Among a multitude of functions, the GM, with its commensal, symbiotic, and mutualistic bacteria, plays a vital role in the dietary nutrient and xenobiotic metabolism of its host, participates in the maintenance of the physiology and structural integrity of the gut mucosa, as well as in bile salt metabolism, vitamin synthesis, and immunomodulation and defence against pathogens.^23–25^ Importantly, several bacterial species produce short-chain fatty acids (SCFA), which exert beneficial effects on humans, with butyrate being a strong anti-inflammatory molecule and the main energy source for enterocytes.^26^ The GM has also been shown to metabolize drugs of cardiovascular interest, including digoxin,^27^ while microbial metabolites correlate with the response to statins.^28^ Indeed, many mechanisms have been proposed by which the GM can affect the bioavailability and actions of various cardiovascular drugs and how these drugs may affect the GM, although, to date, sufficient knowledge of these processes is still lacking.^29^ Even though the human GM remains relatively stable throughout a person’s adult life, compositional intra- and inter-individual variability are considerable and can be influenced by host genetics, geographic origin, age, early life antibiotic use, as well as several other factors, most importantly dietary habits.^30–36^ Population-level studies have attempted to explain the variation that characterizes the human GM. A large metagenomics study from the Netherlands associated part of the variation with a total of 126 factors, both environmental and host-releated.^37^ Most of these factors, however, had a weak effect on microbial composition and diversity. A combination of the database of this study with data from a Belgian population described a core GM shared by 95% of all subjects, yielded further insights, and provided evidence that medication received by the host explains a significant part of the total microbial variation.^38^ Surprisingly, the core GM consisted of less than 20 genera, underscoring the enormous inter-individual variation.

Host genetics can affect the composition of the GM, and colonization experiments have indicated that some host-microbiota metabolic interactions are transferrable traits. For example, genetic aberration leading to absence of the bile acid-sensing Farnesoid X nuclear receptor is responsible for GM-dependent, diet-induced obesity in germ-free mice.^39^ Likewise, early-life, transient antibiotic therapy may provoke significant changes in GM composition, depending on the composition of the microbial community and the antimicrobial spectrum of the antibiotics used, with prolonged consequences for the host.^40–42^ However, a recent randomized controlled trial in preterm infants contradicts this hypothesis.^43^

Host diet is considered to be one of the primary determinants of GM composition.^31,36^ Western diet has been associated with decreased gut microbial diversity, compared to non-Western dietary habits.^31^ The beneficial effects of the Mediterranean diet are partly attributed to the fact that consumption of fruits, vegetables, and legumes, rich in fibre that is processed by bacteria, has been associated with increased SCFA levels. High fibre diets have been proposed to be associated with the largest benefit, due to their association with higher levels of SCFA and increased bacterial diversity.^31^

### 4.2 The pathophysiological basis of gut microbial associations with CAD

Compositional and functional changes of the GM relative to an–arbitrary–healthy state constitute what has been called dysbiosis and can generate CAD-related risk factors, in what has come to be known as the ‘gut-heart axis’. An overview of the pathophysiology will be provided here. The evidence from the relevant animal and human studies are presented in the next section.

Interactions between the GM and components of the cardiovascular system are mainly mediated by bacterial metabolites absorbed by the gut, bacterial molecular signals that can affect host cellular functions, and GM-derived compounds that leak into the systemic circulation (*Table 3*).^44,45^ GM-mediated systemic inflammation may be the main driver of the influence that gut bacteria exert on atherosclerosis and subsequent CAD.^45,46^ Inflammatory pathways are partly mediated by Toll-like receptor (TLR) activity, but although convincing data causally linking inflammation with atherosclerosis exist in rodents, the evidence in humans is scarce.^45^

**Table 3:** Pathophysiological links between the gut microbiota and CAD

Process	Molecules
Infections cause inflammation that may affect atherosclerotic plaque stability.	Cytokines, including tumour necrosis factor-α (TNF-α).
Lipopolysaccharides leaking into the bloodstream trigger low-grade systemic inflammation.	Lipopolysaccharides, TLRs.
Bacterial metabolites may affect proatherogenic celullar functions through foam cell formation and accumulation.	Trimethylamine N-Oxide (TMAO).
Reduced production of anti-inflammatory short-chain fatty acids or indole derivatives may promote systemic inflammation or metabolic dysregulation.	Propionate, acetate, butyrate, tryptophan, indole, indole derivatives.
GM-mediated aberrations in lipid metabolism might influence atherosclerosis.	Bile acids, cholesterol.

Metabolites produced by the GM have emerged as pivotal regulators of signalling pathways directly involved in atherosclerosis and arterial thrombosis. TMAO, produced by the liver from gut microbial TMA, has received considerable attention as a potential key player in the ‘gut-heart axis’, mechanistically linked to atherosclerosis progression by potentially promoting foam cell production and accumulation.^5^ Tryptophan metabolism by gut bacteria has been linked to CVD, through GM-mediated production of indole derivatives. These molecules are mostly beneficial, stimulating production of IL-22 in the gut and possibly strengthening the epithelial barrier.^47,48^ A reduced capacity of the GM to metabolize tryptophan has been associated with host metabolic dysregulation.^49^ Certain conditions, including obesity, inhibit GM-mediated production of indole metabolites, by enhancing tryptophan metabolism towards kynurenine, through increased activity of indoleamine 2,3-dioxygenase (IDO).^50^ Moreover, SCFAs are very important molecules with multiple links to human physiological functions. In general, they are considered beneficial metabolites, associated, among others, with energy production that benefits the host, lipid regulation, immune system modulation, and cardioprotective effects such as beneficial effects of against hypertension-induced cardiac injury and vascular remodelling and atheroprotective properties;^51–56^ these are mediated either directly or via signalling pathways involving G protein-coupled receptors, such as GPR41and GPR43, and the peroxisome proliferator-activated receptors. Lower levels of SCFA are thus likely implicated in aberrations of host physiology.

Other studies have also highlighted the modulation of host lipid metabolism by the GM.^57–59^ Existing evidence links this modulation not only to SCFA but to bile acid signalling as well,^60^ through the bile acid receptor, alternatively known as the Farnesoid X receptor. Bile acid deconjugation in the intestines is known to be mediated by gut bacteria, which also generate secondary bile acids.

The impact of the GM on CAD is not likely restricted to metabolic profiles, as studies have revealed the presence of bacterial DNA in atherosclerotic plaques, implying that gut bacteria could translocate to such lesions to potentially influence plaque inflammatory status and stability.^61,62^

Finally, gut microbial components can trigger low-grade inflammation in humans.^63–65^ LPS from Gram-negative bacteria can leak into the circulation via a disrupted or even intact intestinal barrier, induce inflammation, and contribute to atherosclerosis. The evidence linking LPS to atherosclerosis has been accumulating for more than two decades, highlighting the detrimental role of endotoxins to human health.^6,66^

## 5. Animal and human studies on the association between GM and CAD

### 5.1 Animal studies

Studies in mice have revealed that genetically linked or diet-induced obesity is accompanied by changes in GM composition as well as by induction of systemic inflammation and vascular dysfunction^67,68^ that could be prevented by intermittent fasting.^67^ Dysregulation of GM-related microRNA functions has been shown to induce obesity in mice and inflammation in white adipose tissue.^69^ Similarly, in swine treated with deoxycorticosterone acetate and fed a diet high in fat, salt, and sugar, the diversity of the GM was reduced and its composition shifted towards bacteria associated with host inflammation that was linked with increased circulating levels of tumour necrosis factor-α.^70^ Such studies reveal the links between GM aberrations and host inflammation, a powerful driver of atherosclerosis and, ultimately, CAD.

To investigate causality between gut dysbiosis and CAD, experimental studies have explored the effects of faecal microbiota transplantation to mice from animals that received a high caloric diet^71^ or from patients with CAD.^72^ Such interventions led to alterations in glucose and bile acid metabolism, immune activation, and vascular dysfunction.^71,72^ In LDL-receptor deficient mice, the presence of GM (as opposed to germ-free counterparts) promoted atherothrombosis in a model of ultrasound-induced plaque rupture.^73^ Similarly, microbiota with pro-inflammatory potential transplanted to germ-depleted, LDL-receptor deficient mice accelerated the atherosclerotic process and reduced SCFA concentrations.^74^ Such studies imply that the GM and its dysbiotic states may contribute to CVD, by promoting inflammation and atherosclerosis.

Importantly, in rats suffering a mechanically induced myocardial infarction, alterations in the GM were paralleled by impairment of the intestinal barrier.^75^ These observations suggest that not only intestinal dysbiosis might lead to CAD, but that in turn CAD could perpetuate intestinal dysbiosis, thereby creating a vicious cycle.

Regarding GM-mediated bile salt metabolism and its role in atherosclerosis, FXR-deficient mice have been shown to develop hypercholesterolaemia.^76^  *Apoe* and *Fxr* double-knockout mice develop larger atherosclerotic lesions than ApoE^−/−^ mice.^77^ However, evidence in humans for a role of bile salt metabolism in atherosclerosis is limited.

In a landmark study, TMAO was causally linked to atherosclerosis in rodents.^5^ Susceptibility to atherosclerosis in mice can even be transmitted from high TMAO-producing, atherosclerosis-prone mice to low TMAO-producing, atherosclerosis-resistant mice via the faecal microbiota.^78^ GM-derived TMA can also directly contribute to platelet hyper-reactivity in a number of platelet agonists to enhance a prothrombotic potential.^79^

Finally, in an elegant study, inhibition or deletion of IDO1 in mice fed with a high fat diet resulted in significant differences in gut microbial composition and shifted host tryptophan metabolism away from the kynurenine pathway and towards GM-mediated generation of indole derivatives, resulting in the production of anti-inflammatory IL-22 and decreased endotoxemia.^50^

### 5.2 Human studies

A growing body of clinical evidence also indicates an important role of the GM in CAD (*Table 4*). Although earlier characterization of atherosclerotic plaque microbial communities had been attempted,^61,80^ Koren *et al*.^62^ were the first to examine both the GM and plaque microbial colonies in atherosclerotic patients. They found that microbial colonies of atherosclerotic plaques and gut bacterial communities have distinct compositional differences. They also reported that the GM of atherosclerotic patients was not significantly different from that of controls, although it is unclear how detailed this comparison was. Other studies, however, have found significant differences in beta diversity or individual taxa between patients and controls.^81–88^ Moreover, metabolic features of the GM have been shown to differ between atherosclerotic patients and controls.^81,84^ Metabolic alterations of the GM in individuals with atherosclerosis suggest a microbiota with a higher inflammatory potential.^84^ Studies have demonstrated a decreased abundance of microbes with capacity for producing butyrate and increased circulating levels of TMAO in atherosclerotic patients.^84,86^

**Table 4:** Human studies on the association of the gut microbiota with atherosclerosis and CAD

Study	Location	Sample size	Age (y) patients/controls	Sex (M, %) patients/controls	Analysis approach	Major findings
Koren *et al*.^62^	Single-centre (Sweden)	15 patients/15 controls	65.7 ± 0.5/70.5 ± 0.5	80%/80%	16S rRNA sequencing of stool and atherosclerotic plaque samples	Plaque and gut communities different in beta diversity but several phylotypes common in the same individual. Patient and control group gut microbiota similar.
Karlsson *et al*.^81^	Single-centre (Sweden)	12 patients/13 controls	67.6 ± 8.6/70.5 ± 0.5	75%/77%	Metagenomics in stool samples	Groups different in beta diversity. *Collinsella*, *Roseburia*, and *Eubacterium* genera statistically different in relative abundance between groups.
Yin *et al*.^83^	Single-centre (China)	322 patients/231 controls	61 ± 19/56 ± 11	68.3%/56.3%	16S rRNA sequencing of stool samples, liquid chromatography/mass spectrometry for TMAO measurement	Alpha diversity bigger in patients, beta diversity different between two subgroups of age- and sex-matched pairs. TMAO levels lower in patients than in controls.
Emoto *et al*.^82^	Single-centre (Japan)	39 CAD patients/30 subjects with CVRF	61.1 ± 9.4/62.1 ± 6.4	85%/77%	T-RFLP in stool samples	Increased Lactobacillales and decreased *Bacteroides* and *Prevotella* in CAD patients.
Jie *et al*.^84^	Single-centre (China)	218 ACVD patients/187 controls	60.9 ± 9.8/60.2 ± 9.8	76%/41%	metagenome-wide association study in stool samples.	The ACVD gut microbiome deviates from the healthy status by increased abundance of *Enterobacteriaceae* and *Streptococcus* spp.
Cui *et al*.^85^	Multi-centre (China)	29 CHD in-hospital patients/35 HV	68.3 ± 9.5/66.1 ± 11.4	51%/66%	16S rRNA sequencing of stool samples	Significant decrease in Bacteroidetes and increase in Firmicutes in faecal samples of CHD patients.
Zhu *et al*.^86^	Single-centre (China)	70 CAD patients/98 HV	63.3/60.1	43%/42%	16S rRNA sequencing of stool samples	*Escherichia*, *Shigella* and *Enterococcus* were enriched in the CAD group, while *Faecalibacterium*, *Subdoligranulum*, *Roseburia*, and *Eubacterium rectale* were decreased with CAD.
Liu *et al*.^87^	Single-centre (China)	161 CAD patients/40 HV	62 [54–70]/55 [49–62]	73%/43%	16S rRNA sequencing of stool samples and serum metabolomics	29 metabolite modules that were separately classified as being positively or negatively correlated with CAD phenotypes, and the bacterial co-abundance group with characteristic changes at different stages of CAD was represented by *Roseburia*, *Klebsiella*, *Clostridium* IV, and *Ruminococcaceae*.
Toya *et al*.^88^	Single-centre (USA)	213 CAD patients/53 BMI-matched controls	64.1 ± 8.6/61.6 ± 10.0	60%/56%	16S rRNA sequencing of stool samples	Decreased richness and evenness of gut microbiome in CAD patients. Decreased abundance of *Lachnospiraceae* NK4B4 group and *Ruminococcus gauvreauii* and increased abundance of *R. gnavus* in CAD patients.

ACVD, atherosclerotic cardiovascular disease; BMI: body mass index; CHD, coronary heart disease; CVRF, cardiovascular risk factor; HV, healthy volunteer; T-RFLP, terminal restriction fragment length polymorphism; n.i.: not indicated.

As previously mentioned,^6,64,65^ endotoxins are known causative factors of low-grade systemic inflammation, a crucial step in the pathway of atherosclerosis. In an important prospective study, patients who smoked and had LPS levels above the 90th percentile faced a three-fold increase of the risk for incident atherosclerosis, independently of vascular risk factors.^6^ In high-risk patients for CVD, increased circulating markers of gut-related inflammation, including LPS-binding protein, carried a two-fold increased risk of adverse cardiovascular outcomes.^89^ Circulating levels of LPS have also been associated with a higher risk of major adverse cardiovascular events in a large cohort of patients with atrial fibrillation.^90^

In a seminal study, Wang *et al*.^5^ reported that intestinal microbes participate in phosphatidylcholine metabolism to produce TMA, which is converted to TMAO by the liver, and that TMAO levels predicted major adverse cardiovascular events over a 3-year follow-up. Much research on TMAO followed that pioneering work. Two recent meta-analyses made a systematic evaluation of the relationship between TMAO plasma levels, mortality, and major adverse cardio- and cerebrovascular events.^91,92^ These studies suggested a direct and concentration-dependent association between TMAO levels and all-cause mortality, regardless of conventional risk factors. Fasting plasma TMAO levels were an independent predictor of a high atherosclerotic burden, as estimated with the SYNTAX score in patients with CAD, and of subclinical myocardial injury as quantified by high sensitivity cardiac troponin T (hs-cTnT).^93^ Further, a study in patients presenting with an acute coronary syndrome showed that rapid quantification of trimethyllysine and TMAO at presentation may provide added prognostic value for identifying patients at risk for either short- or long-term adverse cardiovascular events, including in patients with negative hs-cTnT levels at baseline.^94^ However, many other studies have failed to show an association of TMAO with CAD or CVD in general.^3,95–99^ Most importantly, a Mendelian randomization analysis has not shown any relation.^100^

## 6. Therapeutic options

### 6.1 Probiotics

Probiotics are ingestible microorganisms that reach the intestinal lumen, where they can play functional roles in host physiology.^101^ The oral administration of adequate amounts of probiotics has been reported to provide cardiovascular benefit.^102^ Potential mechanisms include the strengthening of gut epithelial tight junctions to reduce LPS leakage and the induction of bile acid deconjugation; this will increase bile acid excretion and force the host to use more cholesterol to counter this effect.

A summary of studies on the antiatherogenic properties of probiotics can be found elsewhere.^103^ As examples, oral administration of the probiotic *Lactobacillus rhamnosus* GR-1 ameliorated cardiac remodelling and pump failure in rats with an acute myocardial infarction produced by a permanent coronary artery occlusion.^104^ Moreover, treating ApoE-deficient mice with *Bacteroides vulgatus* and *B. dorei*, two species that may have a lower abundance in CAD patients, inhibited the formation of atherosclerotic plaques.^105^ Consumption of *L. acidophilus* preparations may have a greater effect in lowering cholesterol than other probiotics.^106^  *Lactobacillus plantarum* ZDY04 significantly reduced plasma TMAO in mice, possibly via remodelling of the GM.^107^

Although preliminary, these studies highlight the therapeutic potential of some probiotic products. However, most positive health claims for probiotics rely on poor evidence, and there are as yet no strong data that probiotic use can prevent CVD. Evidence in humans is scarce and strain-specific effects remain largely unclear.^108^

### 6.2 Prebiotics

Prebiotics are defined as indigestible food components, such as inulins, that promote the growth and/or activity of specific microorganisms within the GM. Conceivably, enhancing the metabolic potential of beneficial bacteria will confer some benefit to host physiology. A summary of animal and human studies of prebiotics can be found elsewhere.^109^ The enhanced production of SCFA and a strengthening of gut epithelial tight junctions are among the potential mechanisms of benefit, although the evidence is less clear in humans. Notably, bacterial metabolite-based interventions (‘postbiotics’) have also been described in the context of intestinal disease, holding promise for broader use.^110^

### 6.3 Synbiotics

Synbiotics are ingestible combinations of probiotics and prebiotics (see definition in the Glossary). A few studies have shown some benefit of synbiotic use in cardiovascular disorders. A summary of human trials can be found in Sáez-Lara *et al*.^111^ As examples, in diabetic patients with CAD, a 12-week intervention with a synbiotic mixture improved glycemic status and HDL levels, but did not alter other cardiovascular risk factors.^112^ Similar studies aimed at reducing hyperglycemia in diabetes had variable results.^113^ In a small randomized, placebo-controlled human trial, the 12-week use of a synbiotic showed a modest reduction in both total cholesterol and low density lipoprotein.^114^ Another randomized controlled trial has also shown benefits in lipid metabolism.^115^ However, the definition of the administered compounds as synbiotics in some trials has been challenged.^116^ Although such results are promising, the most effective synbiotic mixture for reduction of cardiovascular risk has yet to be identified.

### 6.4 Antibiotics

Given the nature of the subject and the abundance of antimicrobial compounds in the medical arsenal, the use of antibiotics has been tested in the context of CAD. However, all relevant trials in the field failed to show any benefit,^117^ and the detrimental effect of broad-spectrum antibiotics to beneficial gut bacteria severely limits this approach.

### 6.5 Dietary interventions

The relationship between diet and human health is well established. A diet characterized by a very low consumption of fibres and a high intake of red meat (or animal proteins), saturated fats, and simple sugars, such as that of Western industrialized societies, has been associated with a high risk of CVD.^118,119^ On the contrary, a nutritional regime characterized by a high consumption of cereals, legumes, nuts, vegetables and fruits, consumption of fish, white meat and eggs, and a low intake of wine, like that of some Mediterranean countries, has been related to a low risk of CVD.^120–124^

As mentioned above, diet has a fast and considerable effect on the composition of the GM.^36^ A summary of the effects that dietary interventions have on the human GM can be found in the studies by Santos-Marcos *et al*.^109^ and Gerdes *et al.*^125^ A dietary pattern rich in fibres may lead to beneficial compositional changes of the GM and higher production of SCFA and may partly explain the benefit derived from the Mediterranean diet.^126,127^ Butyrate in particular has strong anti-inflammatory properties and is considered highly beneficial for human health, providing the main energy source for colonocytes, as already mentioned. Low fibre intake leads not only to a reduction in GM composition and diversity but may also lead to a reduction in the production of SCFA.^128^ At least in rodents, diet rich in saturated fat can increase gut permeability and result in local increase of inflammatory cytokines.^129^ Thus, targeting the interactions between the host and gut microorganisms through change of dietary patterns may lead to prevention of CVD.

### 6.6 Additional interventions that modulate GM–host interactions

Multiple interventions aimed at skewing the profile of the GM towards a more host-beneficial state have been tested. These range from untargeted approaches, such as physical exercise and faecal transplantation, to targeted approaches that include bacterial engineering and drugs affecting bacterial metabolism.^3^ Most of these interventions remain far from reaching clinical practice.

Increased physical activity in humans has been linked to an enhanced bacterial potential for production of SCFA.^130^ The influence of exercise has been reviewed elsewhere.^125^ Faecal transplantation to human recipients, although of medical value in certain pathological conditions like *Clostridioides difficile* infection,^131^ currently lacks robust evidence for a connection to cardiovascular health.^3^ There are however hints of a benefit, as one small study showed that the transfer of GM from lean donors to subjects with metabolic syndrome improved insulin sensitivity and increased GM diversity.^132^ Moreover, in animal models, genetically modified microorganisms may have a therapeutic effect regarding the spectrum of CVD.^133^

Given the numerous studies mentioned above that link elevated TMAO levels with CVD, efforts have been made to inhibit TMAO production.^134–137^ Non-lethal (to microbes) small molecule drugs have been developed to inhibit bacterial trimethylamine lyase systems and have been experimentally tested in animal models, with promising results.

Finally, preliminary approaches in the field of nanomedicine have been conceptualized for future use, to modulate the GM composition, e.g. by using nanoparticles to deliver to the host specific microorganisms associated with favourable metabolic profiles.^30^

## 7. Critical interpretation of published data and methodological challenges

Understanding the role of the GM in cardiovascular risk and CAD has several diagnostic as well as therapeutic implications. Efforts to explore the GM and develop significant clinical applications depend on accurate analyses of microbial communities. Unfortunately, GM studies are confounded by the complexity of microbiome measurements^11^ and are very heterogenous in study design, employed methods, sampling, preservation, measured parameters, and study populations. Such differences limit the ability to reproduce and compare study results, as well as to extrapolate findings to other patient populations. Most importantly, there is yet no universally accepted consensus on what constitutes a healthy GM, and interindividual variability is enormous. Hence, the presence of control subjects in any given study is an *a priori* necessity.

Studies on GM are also often underpowered to capture the substantial variation in the gut microbiome, lack positive and negative controls, and parallel plasma and/or serum samples, making it difficult to translate the functional alterations of the GM to a final impact on health that is reflected in circulating, microbiota-dependent metabolites.^11,12^

Moreover, in most studies, the composition of the GM is typically determined by amplicon sequencing (marker gene analysis), but this approach only yields genus level taxonomic profiles,^9^ lacking any data on bacterial metabolic genes, and is no longer the method of choice.^11^ Various procedural steps in the microbial analysis pipeline of such studies can also be responsible for divergent results between them, as there is no detailed consensus on how to analyse the gut microbiome. A recent study comparing six different approaches reported that DNA extraction methods had the highest impact on observed microbiome variability.^138^ Similarly, a study comparing the European Metagenomics of the Human Intestinal Tract and the American Human Microbiome Project reported significant differences in distribution of bacterial taxa depending on the DNA extraction method.^139^ These results emphasize the risks of comparing data across studies that apply different methodologies and reinforce the need for well-designed longitudinal studies and randomized clinical trials that provide parallel microbiota and plasma/serum samples and are controlled for critical covariates.

Indeed, many studies on the human GM lack the incorporation or statistical handling of key covariates, such as sex, age, body mass index, diet, lifestyle, ethnicity, geographical region, comorbidities, and medication, that could contribute to CVD development and progression, as well as affect the composition of the GM itself.^12,34,35,140–142^ Of note, the use of commonly prescribed drugs such as metformin or proton pump inhibitors leads to changes in the GM,^140,143,144^ and their contribution to compositional variance must be disentangled and accounted for.

Furthermore, the differences between gut microbial communities in humans and rodents hinder the extrapolation of animal study results to human subjects.^145^ Thus, while the analysis of gut microbial composition is a powerful research tool, it remains a complicated process, being relatively far from the point of clinical implementation.

The use of GM metabolites as biomarkers to identify high- and low-risk populations can be more easily implemented.^146^ This may be applicable to widely studied markers such as TMAO, uremic toxins (p-cresol sulphate; indoxyl sulphate—which are particularly significant in the context of chronic kidney disease), bile acids and SCFAs, as well as LPS and other bacterial wall constituents.^147,148^ Nevertheless, integrating multi-omics data is inherently difficult and identifying a metabolite from the microbiome is particularly challenging, as is identifying which microorganism or group of microorganisms is linked to alterations of the levels of a particular metabolite.

Interest in TMAO as a biomarker seems justified given that in healthy subjects, it was associated with a risk of myocardial infarction, stroke and death over a 3-year follow-up.^149^ Also, increased TMAO predicted outcomes even in the presence of other cardiovascular syndromes, such as peripheral artery disease,^150^ and heart failure.^151,152^ TMAO also has prothrombotic and proatherogenic effects,^5,153^ that are not avoided by the platelet aggregation inhibitor ticagrelor.^154^ However, TMAO as a biomarker remains controversial and debated,^95–99^ as conflicting results exist and clear-cut causality in humans has not been proven.

Lastly, as described in the related sections, a number of studies have focused on discerning the effects of GM modulation on cardiovascular health. Although promising evidence exists from research on probiotics and related compounds, this field is still far away from clinical implementation, and more, large, randomized controlled trials are required.

## 8. Conclusion

In a famous philosophical assay published in 1862, Ludwig Feuerbach wrote that we are what we eat. This extreme statement has since been challenged by many other philosophers. While what we eat certainly influences health, it is also not entirely correct, as we are also influenced by GM-dependent interpretation of our diet, which in turn affects various aspects of human physiology and plays a role in different human diseases, spanning from Parkinson’s disease to cancer. Ample evidence suggests a link between the GM and CAD, and while experimental investigations and association studies in human patients suggest that the link may be causal, interventional studies that alter the composition or function of the GM to influence the risk of disease are still lacking. Nevertheless, the information already available indicates that markers of gut bacterial dysbiosis may improve the risk stratification for CAD. The interest in this new human ‘organ’ and our ‘second genome’ is escalating fast.
